# Mutation and clinical analysis of the *CLCC1* gene in amyotrophic lateral sclerosis patients from Central South China

**DOI:** 10.1002/acn3.51934

**Published:** 2023-11-02

**Authors:** Linxin Tang, Xuxiong Tang, Qianqian Zhao, Yongchao Li, Yue Bu, Zhen Liu, Jinchen Li, Jifeng Guo, Lu Shen, Hong Jiang, Beisha Tang, Renshi Xu, Wenfeng Cao, Yanchun Yuan, Junling Wang

**Affiliations:** ^1^ Department of Neurology, Xiangya Hospital Central South University, Jiangxi Hospital, National Regional Center for Neurological Diseases Nanchang P. R. China; ^2^ Department of Neurology, Xiangya Hospital Central South University Changsha P. R. China; ^3^ National Clinical Research Center for Geriatric Diseases, Xiangya Hospital Central South University Changsha P. R. China; ^4^ Center for Medical Genetics, School of Life Sciences Central South University Changsha P. R. China; ^5^ Key Laboratory of Hunan Province in Neurodegenerative Disorders Central South University Changsha P. R. China; ^6^ Engineering Research Center of Hunan Province in Cognitive Impairment Disorders Central South University Changsha P. R. China; ^7^ Hunan International Scientific and Technological Cooperation Base of Neurodegenerative and Neurogenetic Diseases Changsha P. R. China; ^8^ Hunan Provincial University Key Laboratory of the Fundamental and Clinical Research on Neurodegenerative Diseases Changsha P. R. China; ^9^ Jiangxi Provincial People's Hospital, Clinical College of Nanchang Medical College First Affiliated Hospital of Nanchang Medical College Nanchang P. R. China

## Abstract

**Introduction:**

Recently, chloride channel CLIC‐like 1 (*CLCC1*) was reported to be a novel ALS‐related gene. We aimed to screen *CLCC1* variants in our ALS cohort and further explore the genotype–phenotype correlation of *CLCC1*‐related ALS.

**Methods:**

We screened rare damaging variants in *CLCC1* from our cohorts of 1005 ALS patients and 1224 healthy controls with whole‐exome sequencing in Central South China. Fisher's exact test was conducted for association analysis at the entire gene level and single variant level.

**Results:**

In total, four heterozygous missense variants in *CLCC1* were identified from four unrelated sporadic ALS patients and predicted to be putative pathogenic by in silico tools and protein model prediction, accounting for 0.40% of all patients (4/1005). The four variants were c.A275C (p.Q92P), c.G1139A (p.R380K), c.C1244T (p.T415M), and c.G1328A (p.R443Q), respectively, which had not been reported in ALS patients previously. Three of four variants were located in exon 10. Patients harboring *CLCC1* variants seemed to share a group of similar clinical features, including earlier age at onset, rapid progression, spinal onset, and vulnerable cognitive status. Statistically, we did not find *CLCC1* to be associated with the risk of ALS at the entire gene level or single variant level.

**Conclusion:**

Our findings further expanded the genetic and clinical spectrum of *CLCC1*‐related ALS and provided more genetic evidence for anion channel involvement in the pathogenesis of ALS, but further investigations are needed to verify our findings.

## Introduction

Amyotrophic lateral sclerosis (ALS) is a fatal neurodegenerative disease characterized by muscle weakness and atrophy due to degeneration of motor neurons.^1^ The number of ALS patients was estimated to increase to 376,674 by 2040.^2^ Genetic factors play important roles in the pathogenesis of this disease.^3^, ^4^, ^5^ Approximately 10% of ALS cases are familial ALS (fALS), and the remaining cases are sporadic ALS (sALS).^2^, ^6^, ^7^ The heritability was estimated to be 38%–78% in twin studies,^8^ 8.5% in large GWAS datasets,^9^ and 52.3% in population registers.^5^ Mutations in major ALS‐related genes account for approximately 47.7% of fALS and 5.2% of sALS.^10^ The missing heritability could be partially explained by unidentified ALS‐related genes. To date, more than 50 genes have been reported to contribute to ALS pathogenesis.^11^, ^12^, ^13^, ^14^ With advanced sequencing and analysis techniques, an increasing number of novel ALS‐related genes have been identified in recent years, such as genes encoding kinesin proteins involved in axonal transport (*KIF5A* ^15^, ^16^ and *KIF1A* ^17^), the apoptosis‐related gene *TP73*,^18^, ^19^ the sphingolipid synthesis‐related gene *SPTLC1*,^20^, ^21^, ^22^ and the fatty acid metabolism‐related gene *ACSL5*.^23^, ^24^, ^25^ The newly discovered genes broaden our overall understanding of ALS pathogenesis.

In a recent study, the gene chloride channel CLIC like 1 (*CLCC1*) was reported to be a novel ALS‐related gene.^26^ He et al. carried out a burden analysis in a Chinese cohort of 670 sALS patients and 1910 controls and revealed that *CLCC1* was associated with ALS.^26^ CLCC1, a pore‐forming component of an anion channel on the endoplasmic reticulum (ER), regulates the unfolded protein response and cellular homeostasis, and maintains constant homeostasis of ER ions.^26^, ^27^ Notably, accumulated misfolded proteins and ER stress are important pathways underlying the pathogenesis of ALS.^11^, ^28^ In a mouse model, ALS‐related *CLCC1* mutations and loss of *CLCC1* disrupted ER ion homeostasis and then led to ALS‐like symptoms and pathologies.^26^, ^29^ In addition to ALS, *CLCC1* has also been reported to be mutated in autosomal recessive retinitis pigmentosa patients.^30^, ^31^ The *CLCC1*‐related phenotype showed clinical and genetic heterogeneity. More studies are required to investigate the genotype–phenotype associations of *CLCC1*.

In this study, we aimed to (1) screen the *CLCC1* mutations in our ALS cohort in Central South China and (2) determine the correlation between clinical manifestations and mutations, further expanding the genetic and clinical spectrum of ALS.

## Materials and Methods

### Subjects

In total, 1005 ALS patients from the central southern region of China were recruited at Xiangya Hospital, Central South University, consisting of 75 fALS and 930 sALS patients. The fALS included AD‐ALS and AR‐ALS. We defined AD‐ALS as ALS diagnosed in family members in at least two consecutive generations, and AR‐ALS as having a consanguineous family history or having at least two family members with ALS symptoms in only one generation. ALS patients were diagnosed by at least two experienced neurologists according to the revised EI Escorial criteria 2015.^32^ As HCs, a total of 1224 neurologic disease‐free individuals of Chinese ancestry matched by geography were collected at the Health Center of Xiangya Hospital. The demographic information of all individuals is listed in Table S1. We also selected 6708 whole‐exome sequencing and 780 whole‐genome sequencing data from East Asian individuals without any neurologic disease in the gnomAD database v2 as a control cohort together in association analysis. This study was approved by the Ethics Committee of Xiangya Hospital, Central South University. Written informed consent was obtained from all individuals.

### Mutation analysis

Peripheral blood samples were collected from all participants, and genomic DNA was extracted by a standard protocol. All patients with ALS underwent whole‐exome sequencing. Sequencing procedures, including quality control and annotation, were the same as previously reported.^33^ The variants were mapped to the GRCh37 reference genome.

Rare damaging variants (RDVs) in *CLCC1* variants were screened by the following criteria: (1) heterozygous or homozygous variants in the *CLCC1* gene; (2) for heterozygous variants, minor allele frequency (MAF) less than 0.1% in 1000 Genomes (1000 Genomes Project), ESP6500s (NHLBI‐ESP project with 6500 exomes), GnomAD (Genome Aggregation Database), and ExAC (Exome Aggregation Consortium); for homozygous variants, MAF less than 1%; (3) absent in our HC cohort; (4) nonsynonymous, indels, frameshift, and putative splice site variants; and (5) pathogenicity defined as predicted to be pathogenic by at least two in silico tools. The in silico prediction tools are listed in Table S2.

As these identified RDVs in *CLCC1*, we also screened them in publicly available ALS databases, including the ALSdb (http://alsdb.org/), Project MinE (http://databrowser.projectmine.com/), and ALS variant server (http://als.umassmed.edu/).

Other known ALS‐related variants were screened in patients harboring *CLCC1* variants by the above standards similar to that of *CLCC1*. *C9orf72* and *ATXN2* repeat expansion mutations were also screened to exclude certain pathogenic variants.^8^, ^33^

### Clinical analysis

A comprehensive battery of clinical data, including age, sex, family history, and clinical features such as age at onset (AAO), site of onset, disease duration, physical examination, MMSE (Mini‐Mental State Examination), ECAS (Edinburgh Cognitive and Behavioral ALS Screen), and ALS Functional Rating Scale–Revised (ALSFRS‐R) score, were collected from all patients. The progression rate (ΔFS) was calculated as ΔFS = (48‐ALSFRS‐R score at “time of diagnosis”)/duration from onset to diagnosis (month).^34^ Survival time was recorded through longitudinal follow‐up analysis.

### Protein structural prediction

A three‐dimensional structural model of wild‐type (WT) and mutated CLCC1 protein was predicted by Alphafold2 colab (AlphaFold2.ipynb), and visualization was conducted by PyMol software (Version 2.5.4). DynaMut2 was used to assess protein stability.^35^

### Statistical analysis

Statistical analysis was performed with SPSS 25.0. Descriptive statistics (mean ± SD) or median (IQR) were calculated for continuous variables according to whether normality distribution was satisfied. The comparison of continuous variables was assessed by Student's *t* test or Mann–Whitney *U* test. For comparison of categorical variables, the chi‐square test was applied. Association analysis of the RDVs was performed across the entire *CLCC1* gene and identified variants using Fisher's exact test. *p* < 0.05 were considered statistically significant.

## Results

### Demographics

In total, we analyzed *CLCC1* variants in 1005 ALS patients and 1224 HCs of Chinese ancestry in our study. In patients, the AAO (median [IQR]) was 54 (47,62) years old, and the age at sampling was 56 (49,63) years old. The geographically matched HCs were older than ALS patients, with a median age of 68 (65,73) years, to avoid recruiting presymptomatic patients. ALS patients with spinal onset accounted for 75.42% (758/1005), while 18.11% (182/1005) had bulbar onset. Notably, 6.47% (65/1005) of patients presented with spinal and bulbar onset almost simultaneously. The detailed demographic characteristics of all individuals are listed in Table S1.

### Mutation analysis of *CLCC1* in the ALS cohort

**Figure 1:**
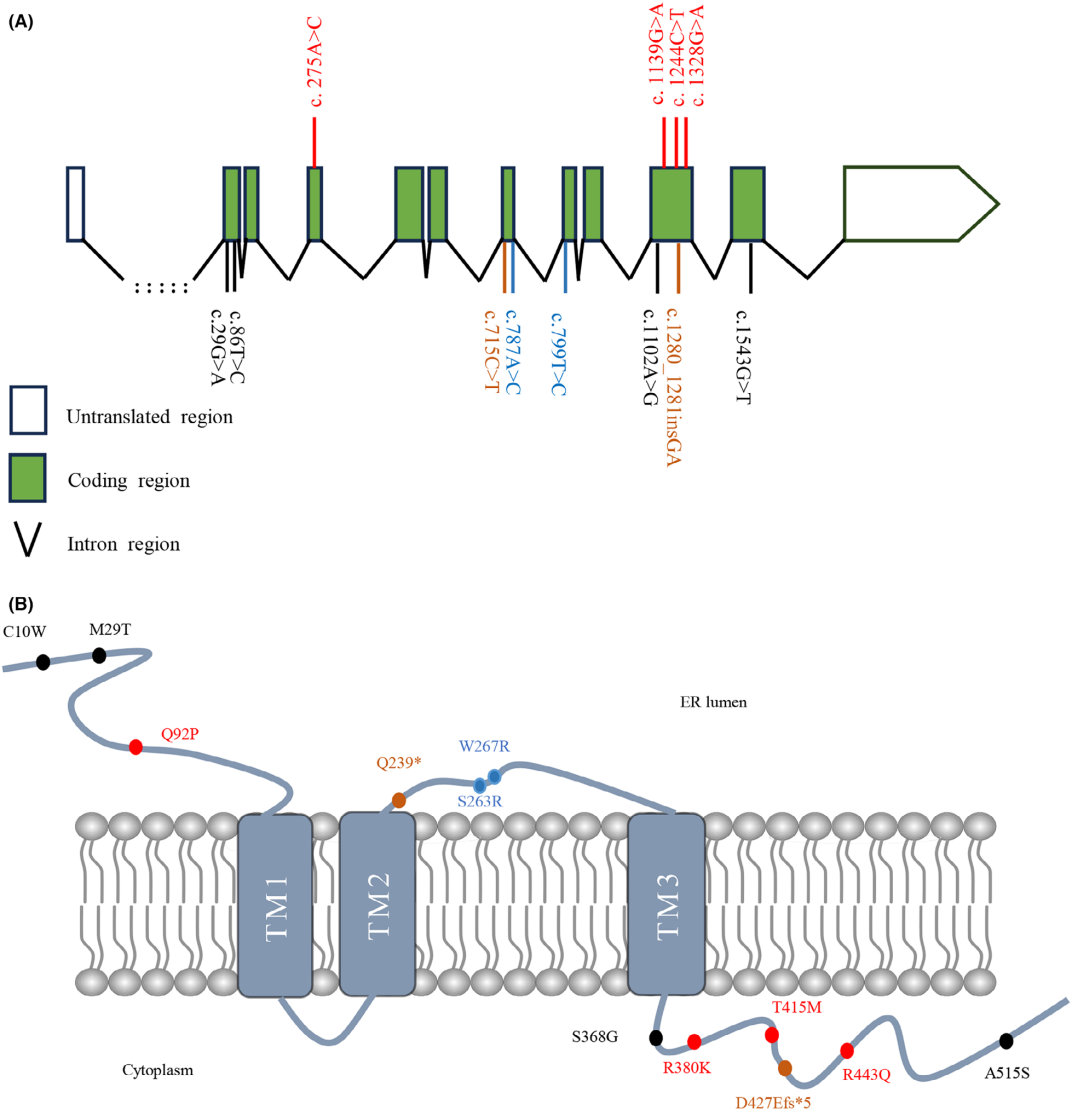
Rare, putative pathogenic variants in *CLCC1* identified in ALS patients. (A) Schematic representation of the *CLCC1* transcript NM_001048210. (B) Schematic representation of the CLCC1 protein. Novel putative pathogenic variants in our ALS cohort (red font); pathogenic variants reported by He et al. with functional experiments supported (blue font); truncated variants reported by He et al. (brown font); other rare putative pathogenic variants reported by He et al. (black font). ER, endoplasmic reticulum; TM, transmembrane.

In our cohort, four heterozygous RDVs in *CLCC1* were identified in four unrelated sALS patients, accounting for 0.40% of all patients (4/1005). Rare homozygous variants were not identified in our cohort. The four heterozygous variants were c.A275C (p.Q92P), c.G1139A (p.R380K), c.C1244T (p.T415M), and c.G1328A (p.R443Q) (Table 1). Three of four variants were located in exon 10, while the remaining variant was in exon 4 (Fig. 1A). Corresponding with the location in exon 10, the amino acid locations of these variants were located on the cytoplasmic side beyond transmembrane domain 3 (Fig. 1B). Among them, the variant c.G1139A was not found in any public genome database, and variants c.A275C (p.Q92P) and c.G1328A (p.R443Q) were predicted to be pathogenic at a relatively high degree of confidence. Specifically, for these four variants—c.275A>C (p.Q92P), c.1139G>A (p.R380K), c.1244C>T (p.T415M), and c.1328G>A (p.R443Q)—eight, three, two, and six out of nine in silico tools, respectively, predicted them as pathogenic (Table S2). The CADD prediction results of these four variants were damaging (20), tolerable (10.66), tolerable (14.87), and damaging (23.6), respectively. And the Polyphen2 HDIV and HVAR predicted all these four variants to be damaging. In addition, variants c.C1244T (p.T415M) and c.G1328A (p.R443Q) were reported in the ALSdb and Project MinE ALS databases, respectively (Table S3).

**Table 1:** *CLCC1* rare damaging variants identified in our ALS cohort.

Sample ID	Chr1	Exon	cDNA change	AA change	Mutation type	dbSNP	HCs	GnomAD_exome_ALL	GnomAD_exome_EAS	ExAC	1000 Genomes	ESP6500s
XY0254	109490297	4	c.A275C	p.Q92P	missense	NA	NA	4.10E‐06	5.85E‐05	NA	NA	NA
XY0736	109479943	10	c.G1139A	p.R380K	missense	NA	NA	NA	NA	NA	NA	NA
XY0010	109479838	10	c.C1244T	p.T415M	missense	rs759574194	NA	2.87E‐05	1.00E‐04	2.50E‐05	NA	NA
XY0098	109479754	10	c.G1328A	p.R443Q	missense	rs758737739	NA	2.11E‐05	6.48E‐05	9.03E‐06	NA	NA

Genome position was based on GRCh37. RefSeq accession number: NM_ 001048210 for *CLCC1*.

Abbreviations: AA, amino acids; ALS, amyotrophic lateral sclerosis; cDNA, complementary DNA; dbSNP, Database of Single Nucleotide Polymorphism; EAS, East Asian; ESP6500s, NHLBI‐ESP project with 6500 exomes; ExAC, Exome Aggregation Consortium; GnomAD, Genome Aggregation Database; HCs: healthy controls; NA, not available; 1000 Genomes, 1000 Genomes Project.

We also screened mutations in other known ALS‐causative genes in these four patients. The patient carrying variant c.A275C (p.Q92P) of *CLCC1* also had one unreported missense mutation in *EWSR1* (NM_005243:c.G1742A:p.R581H).

### Results of association analysis at the *CLCC1* gene level and single variant level

In this study, we enrolled two cohorts of controls, our HC cohort and East Asian individuals without any neurologic disease in the gnomAD database v2. Compared with controls, we did not find RDVs in the *CLCC1* gene overexpressed in ALS patients at the entire gene level (Table 2). Moreover, Fisher's exact test found that no variant was associated with the risk of ALS (Table 2).

**Table 2:** Association analysis results of the identified rare damaging variants in *CLCC1.*

Model	AC (patients) *n* = 1005	HCs, *n* = 1224			GnomAD_EAS_non_neuro, *n* = 7488^a^		
		AC	*p* value	OR [95% CI]	AC	*p* value	OR [95% CI]
Gene level	4	5	1.00	0.97 [0.30–3.21]	33	1.00	0.90 [0.34–2.38]
c.A275C	1	0	0.45	Inf [0.14‐Inf]	0	0.12	Inf [0.83‐Inf]
c.G1139A	1	0	0.45	Inf [0.14‐Inf]	1	0.22	7.45 [0.40–141.60]
c.C1244T	1	0	0.45	Inf [0.14‐Inf]	2	0.31	3.73 [0.26–32.07]
c.G1328A	1	0	0.45	Inf [0.14‐Inf]	0	0.12	Inf [0.83‐Inf]

Abbreviations: AC: allele account; CI, confidence interval; EAS, East Asian; GnomAD, Genome Aggregation Database; HCs: healthy controls; Inf: Infinity; NA: not available; neuro: neurologic disease; OR, odds ratio.

^a^ GnomAD_EAS_non_neuro included 6708 WES data and 780 WGS data.

### Clinical characteristics of ALS patients with variants in *CLCC1*

Summarizing the clinical manifestations of the four ALS patients carrying RDVs in *CLCC1*, we found that these four patients shared relatively similar clinical features. The mean AAO of the four patients was 48.50 ± 2.87 years, ranging from 40 to 52 years. All four patients showed spinal onset. The disease progressed rapidly, with the progression rates ΔFS greater than 0.75, and an average ALSFRS‐R score of 41. The survival time ranged from 13 to 30 months. Interestingly, we found that these patients tended to display cognitive decline (50.0%, 2/4). The clinical features of patients with variants in *CLCC1* are listed in Table 3.

**Table 3:** Clinical manifestations of ALS patients with rare damaging variants in *CLCC1.*

Patients	This study				He et al.
	Patient 1	Patient 2	Patient 3	Patient 4	
Sample ID	XY0254	XY0736	XY0010	XY0398	NA
Variants	c.A275C	c.G1139A	c.C1244T	c.G1328A	NL
Family history	Sporadic	Sporadic	Sporadic	Sporadic	Sporadic
Sex	Female	Female	Female	Male	Female (3)
					Male (6)
Age at onset	52	50	52	40	45 (34, 74)^a^
Alive (Y/N)	N	N	N	NA	NL
Survival (months)	21	13	30	NA	78 (13)^b^
Site of onset	Right distal upper limb	Left distal upper limb	Right upper limb	Left distal upper limb	Hand (4)
					Lower limb (5)
Muscle weakness and atrophy	Generalized	Generalized	Upper limbs	Left limb	NA
Reflexes	Hyporeflexia	Hyperreflexia	Hyperreflexia	Hyperreflexia in upper limbs	NA
Bulbar involved	Y	N	Y	N	NA
ALSFRS‐R	40/48	38/48	39/48	47/48	NA
ΔFS	1.60	1.11	0.75	1.00	NA
Education level (years)	9	9	12	9	NA
MMSE	NA	NA	22/30	30/30	NA
ECAS	71/136	NA	NA	NA	NA
EMG	OD and CR in all four segments	OD and CR in all four segments	OD and CR in cervical and lumbar segments	OD and CR in bulbar, cervical, and lumbar segments	NA

Abbreviations: ALS, amyotrophic lateral sclerosis; ALSFRS‐R, ALS functional rating scale revised; CR, chronic reinnervation; ECAS, Edinburgh Cognitive and Behavioral ALS Screen; EMG, electromyogram; MMSE, Mini‐Mental State Examination; N, no; NA, not available; NL, not listed; OD, ongoing denervation; PMA, progressive muscular atrophy; Y, yes; ΔFS, progression rate, calculated as (48‐ALSFRS‐R at “time of diagnosis”)/duration from onset to diagnosis (month).

^a^ Median (IQR).

^b^ Mean (SD).

P1 (XY0254) was a female carrying variant c.A275C (p.Q92P), characterized by rapid regressive muscle weakness of four limbs and early involvement of the bulbar region. Starting from weakness of the right hand at the age of 52 years, the left upper limb and lower limbs became involved within 3 months, gradually manifesting obvious muscular atrophy of the four limbs. Four months after onset, the patient showed dysarthria without obvious dysphagia. At the time of visit of 5 months since onset, her ALSFRS‐R score was 40/48. Her tendon reflexes were decreased, and no pathological reflex was elicited. An electromyogram (EMG) test showed widespread ongoing denervation and chronic reinnervation of all four segments (bulbar, cervical, thoracic, and lumbosacral). Diagnosed with PMA, this patient did not present any signs of upper motor neuron (UMN) degeneration. She died 21 months after the onset of symptoms.

P2 (XY0736) was a female harboring variant c. G1139A (p.R380K). At the age of 50, she initially presented with weakness and atrophy of the left upper limb, which spread to the right upper limb and lower limbs within 6 months. She developed dysarthria 8 months later. Nine months later, at the first visit, her ALSFRS‐R score was 38/48. Muscle fasciculations were observed in all four limbs. The deep tendon reflexes of the lower limbs were brisk, and palmomental reflexes were positive. The ECAS screen showed naming impairment and executive dysfunction (total score: 71/136, naming domain: 0/8, executive function: 20/48). EMG showed classic widespread chronic reinnervation and ongoing denervation in all four segments. Subsequent axonal damage of peripheral nerves was also observed. She was diagnosed with probable ALS and died of respiratory failure 13 months later.

P3 (XY0010), a female with variant c.C1244T (p.T415M), showed initial symptoms of muscle weakness and atrophy of the right upper limb at 52 years old. Six months later, she complained of memory impairment. Nine months later, her left upper limb was involved. Almost 11 months later, she presented with dysphasia. Twelve months later, at the first visit, her ALSFRS‐R score was 39/48. MMSE showed deficiency mainly in executive and language functions. EMG revealed axonal damage of nerves of the right upper limb as well as ongoing denervation and chronic reinnervation of cervical and lumbosacral regions. Her diagnosis was probable ALS. Nearly 28 months later, at our follow‐up, weakness of her upper limbs deteriorated, and dysphagia developed. The patient died 30 months later.

P4 (XY0398) was a male with variant c.G1328A (p.R443Q) who complained of weakness and atrophy of the left upper limb at 40 years old. He visited 1 month later since onset, and his ALSFRS‐R score was 47/48. His tendon reflexes of the upper limbs were brisk with a positive Hoffmann sign on the left side. Obvious muscular atrophy was observed in the left upper limb. EMG showed ongoing denervation and chronic reinnervation in bulbar, cervical, and lumbosacral segments. His clinical diagnosis was probable ALS laboratory supported. Unfortunately, we lost touch with him in later follow‐up.

### Generation and stability analysis of 3D models of CLCC1 protein

**Figure 2:**
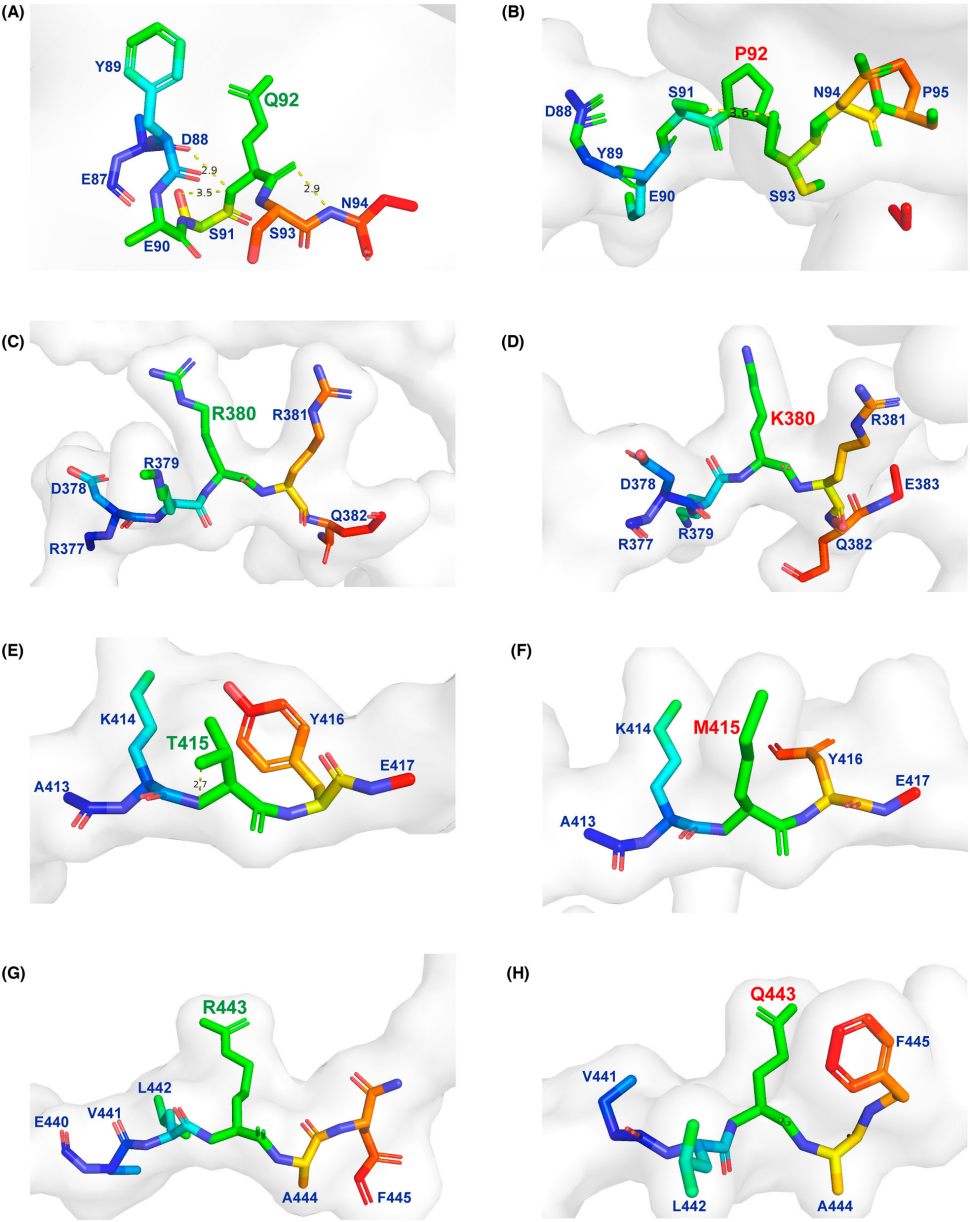
3D structural prediction models of CLCC1 protein. (A and B) Structure of the 92nd position in wild‐type and mutated CLCC1 (p.Q92P). (C and D) Structure of the 380th position in wild‐type and mutated CLCC1 (p.R380K). (E and F) Structure of the 415th position in wild‐type and mutated CLCC1 (p.T415M). (G and H) Structure of the 443rd position in wild‐type and mutated CLCC1 (p.R443Q). Wild‐type amino acid residues are identified in green labels, mutated amino acid residues are identified in red labels, neighboring amino acid residues are identified in blue labels, hydrogen bonds are identified in yellow dashed lines, and the number above the dashed line is the predicted hydrogen bond length.

We constructed 3D structural models of the WT and mutated CLCC1 proteins (Fig. 2) and assessed their stabilities. In the first model, the amino acid at the 92nd position was converted from a polar amino acid Gln to a nonpolar Pro, and the hydrogen bonds between site 92 and Asn 94 or Asp 88 were disrupted, while hydrogen bonds between site 92 and Ser 91 still existed. In the second model where Lys substituted for Arg at site 380, no hydrogen bond was found in either the predicted WT or mutated protein when the distance was extended to 8 Å. However, DynaMut2 analysis predicted that the variant would decrease the stability of the CLCC1 protein due to the negative free energy values (ΔΔG = −0.49 kcal/mol). The third variant, converting Thr to Met, disrupted the hydrogen bond at site 415, and the mutated protein also showed predicted decreased stability (ΔΔG = −0.45 kcal/mol). In the fourth model, where positively charged Arg 443 was replaced with Gln with no charge, there were no surrounding hydrogen bonds at a distance of 8 Å, but the variant may destabilize the CLCC1 protein (ΔΔG = −0.17 kcal/mol). These altered hydrogen bonds and decreased stabilities implied potential changes in protein function and interaction.

## Discussion

CLCC1 is a transmembrane protein localized on the ER. It contributes to anion transport between the ER and cytoplasm and maintains ionic homeostasis.^26^ Dysfunction of CLCC1 causes misfolding of proteins in the ER.^27^, ^29^ Reportedly, the c.75C>A (p.D25E) mutation was associated with autosomal‐recessive retinitis pigmentosa in a Pakistani population.^30^, ^31^ Recently, *CLCC1* was reported to be a novel ALS‐related gene, and its functional mutation and loss of *CLCC1* caused an ALS‐like phenotype in mice.^26^ This finding provides new insight into the pathogenesis underlying ALS. This study is the first to propose the role of disrupted ER ion homeostasis maintained by an ER anion channel in ALS pathogenesis. Interestingly, the ALS‐related variants (including missense and truncated variants) were heterozygous in this recent study.^26^ The reported ALS‐related variants decreased the protein level of CLCC1 compared with WT. In vivo, the severity of the ALS‐like phenotype varied in a pattern of CLCC1 dosage dependence.^26^ Clinically, the patients with mutations in *CLCC1* had high heterogeneity.

In our ALS cohort from Central South China, we discovered four heterozygous RDVs in *CLCC1* in four unrelated sALS patients. Our findings broaden the current genetic and clinical spectrum of *CLCC1* in ALS and provide clues for further investigation. The four identified rare missense variants were not the same as the variants reported by He et al.^26^ In silico prediction and 3D models suggested their potential pathogenicity. Among them, variant c.G1139A (p.R380K) has not been found in any public genome database. Another two heterozygous variants, c.C1244T (p.T415M) and c.G1328A (p.R443Q), were reported in the ALSdb and Project MinE ALS databases, respectively. As detailed clinical information was not accessible, we cannot include the clinical features of the two variants in the public ALS database in the analysis.

Similar to previous reports,^26^ the variants identified in this study were also not located in transmembrane regions (Fig. 1). The variants we found were concentrated at exon 10, indicating that exon 10 might be a potential hotspot. To verify this hypothesis, we analyzed the distribution of rare variants of *CLCC1* in HCs. Five rare variants were identified in HCs according to the same criteria as in ALS patients except the third criterion (Table S4). There was no tendency of centralization in rare variants identified in HCs, and only one variant was located in exon 10. However, there was no significant difference in the frequency of rare variants within exon 10 between patients and HCs (*p* = 0.21). Consistently, three of four variants in ALS patients localized at the C‐terminus, while previously reported variants were mostly centralized at the N‐terminus.^26^ The variants at the C‐terminus in our study were predicted to affect protein stability. As reported, the N‐terminal variants (K298A, S263R, and W267R) in heterozygous status were as stable as WT CLCC1, while C‐terminal‐truncated mutations might affect full‐length CLCC1 protein stability.^26^ Further experiments are required to explore whether the C‐terminal structure is significant for maintaining protein stability and its underlying mechanism.

To summarize the clinical manifestations of *CLCC1*‐related ALS patients in our cohort, it seemed that they showed relatively severe clinical manifestations, such as earlier AAO, rapid progression, and short survival time. In addition, all patients showed spinal onset, and half of the patients had cognitive impairment. First, the *CLCC1*‐related ALS patients showed an earlier AAO (48.50 ± 2.87 years, ranging from 40 to 52 years), while our previous work reported a mean AAO of 54.3 ± 10.9 years in sALS patients.^33^ Second, the *CLCC1*‐related patients had short survival times, ranging from 13 to 30 months, except for the patient loss to follow‐up. Moreover, our patients showed relatively rapid progression with a ΔFS greater than 0.75. Consistently, *CLCC1*‐related ALS patients in this study showed similar spinal onset as ALS patients reported by He et al. Last, two patients in our study manifested cognitive decline mainly in language and executive domains, which are relatively vulnerable in ALS patients.^36^, ^37^ However, patients harboring *CLCC1* variants also showed high heterogeneity clinically. The AAO of *CLCC1* patients reported by He et al. ranged from 17 to 74 years, and the survival time ranged from 38 to 135 months.^26^ Notably, the sample size in this study was limited, and therefore, larger cohorts are needed to validate the above hypotheses.

In this study, we also provided evidence for the oligogenic model of ALS.^4^, ^38^, ^39^, ^40^ In our cohort, patient XY0254 with variant c.A275C (p.Q92P) also harbored an additional variant in another known ALS‐related gene, c.G1742A (p.R581H) in *EWSR1*. The AAO of this patient was 52 years old. The survival time was 21 months with a ΔFS of 1.60. The frequency of this variant in *EWSR1* in the public databases was less than 0.01%, and functional prediction was predicted pathogenic by six out of nine in silico tools. We presumed that the two variants might develop a synergistic effect on the phenotype. As reported, patients carrying more RDVs tended to have an earlier AAO and a more rapid progression.^4^, ^38^, ^41^, ^42^

In summary, we screened the rare variants of the recently discovered ALS‐related *CLCC1* gene in our ALS cohort from Central South China and found four putative pathogenic variants. The clinical characteristics of *CLCC1*‐related patients included spinal onset, relatively rapid progression, and possible cognitive decline. Our findings expand the genetic and clinical spectrum of *CLCC1*‐related ALS and provide more genetic evidence for the pathogenesis related to ion channels and clues for further investigation.

## Author Contributions

Linxin Tang performed the majority of the analyses and drafted the article. Xuxiong Tang was responsible for the structural prediction analysis. Qianqian Zhao, Yongchao Li, and Yue Bu contributed to the collection of clinical data. Zhen Liu and Jinchen Li contributed to bioinformatic analyses. Jifeng Guo, Lu Shen, Hong Jiang, Beisha Tang, Renxu, Xu, Wenfeng Cao, and Junling Wang provided critical intellectual contributions and participated in article revision. Yanchun Yuan was responsible for the study concept and design and writing the article.

## Disclosures

The authors declare no competing financial interests.

## Funding Information

This work was supported by the Science and Technology Innovation 2030 (STI2030‐Major Projects: 2021ZD0201803 to J.W.); the National Key R&D Program of China (No. 2021YFA0805202); the Program of the National Natural Science Foundation of China (#82171431, 81671120, and 81300981 to J.W.); and the Project Program of National Clinical Research Center for Geriatric Disorders at Xiangya Hospital (#2020LNJJ13 to J.W.).

## Supporting information


Supplementary Table 1.
