# Supplementary material for: Mutation and clinical analysis of the CLCC1 gene in amyotrophic lateral sclerosis patients from Central South China
Source: Ann Clin Transl Neurol. 2023 Nov 2;11(1):79–88. doi: 10.1002/acn3.51934 (PMC10791024; doi:10.1002/acn3.51934)
Supplement: Supplementary file 1 — Supplementary Table 1. [file ACN3-11-79-s001.docx]

**Supplementary Table 1. Demographic characteristics of ALS patients and HCs**

|  | ALS patients | HCs |
| --- | --- | --- |
| Numbers | 1,005 | 1,224 |
| Family history, n | 75 | NA |
| Male, n (%) | 652 (64.88) | 577 (47.14) |
| Age at onset, y | 54 (47, 62) | NA |
| Age at sampling, y | 56 (49, 63) | 68 (65, 73) |
| Spinal onset, n (%) | 758 (75.42) | NA |
| Bulbar onset, n (%) | 182 (18.11) | NA |
| Spinal & bulbar onset, n (%) | 65 (6.47) | NA |

***Abbreviations:*** ALS, amyotrophic lateral sclerosis; HCs: healthy controls; NA, data not available.

**Supplementary table 2. Function predictions of the identified rare damaging variants in *CLCC1* by in-silico tools**

| **Variants** | **CADD** | **DANN** | **MCAP** | **Mutation Taster** | **Mutation Assessor** | **Polyphen2 HDIV** | **Polyphen2 HVAR** | **SIFT** | **VEST3** | **Total** |
| --- | --- | --- | --- | --- | --- | --- | --- | --- | --- | --- |
| c.275A>C  (p.Q92P) | Damaging  (20.000) | Damaging (0.996) | Damaging (0.030) | Disease causing (1.000) | Medium (2.915) | Probably D  (0.999) | Probably D (0.974) | Tolerable (0.089) | Damaging (0.827) | 8 (9) |
| c.1139G>A (p.R380K) | Tolerable (10.660） | Tolerable (0.985) | Tolerable (0.015) | Polymorphism (1.000) | Medium (2.825) | Probably D  (0.986) | Possibly D (0.694) | Tolerable (0.518) | Tolerable (0.222) | 3 (9) |
| c.1244C>T (p.T415M) | Tolerable (14.870) | Tolerable (0.88) | Tolerable (0.022) | Polymorphism (1.000) | Low  (1.505) | Probably D  (0.965) | Possibly D (0.503) | Tolerable (0.219) | Tolerable (0.121) | 2 (9) |
| c.1328G>A (p.R443Q) | Damaging (23.600) | Damaging (0.998) | Tolerable (0.017) | Disease causing (1.000) | Medium  (1.990) | Probably D  (0.992) | Possibly D (0.608) | Tolerable (0.134) | Tolerable (0.147) | 6 (9) |

***Abbreviations:*** CADD, Combined annotation dependent depletion; DANN, Deleterious annotation of genetic variants using neural networks. D, damaging; MCAP, Mendelian clinically applicable pathogenicity; PolyPhen2 HDIV, Polymorphism Phenotyping Version 2 Human Diversity; PolyPhen2 HVAR, Polymorphism Phenotyping Version 2 Human Variation; SIFT, Sorting Intolerant From Tolerant; VEST, Variant Effect Scoring Tool.

**Supplementary table 3. Variants in CLCC1 identified in publicly available ALS databases**

| Database | Chr1 | Variant | Allele Count | Total Sample Count |
| --- | --- | --- | --- | --- |
| ALSdb | 109479838 | c.C1244T: p.T415M | 1 | 3,317 |
| Project MinE | 109479754 | c.G1328A: p.R443Q | 1 | 4,366 |

Genome position was based on GRCh37. RefSeq accession number: NM_ 001048210 for *CLCC1*.

**Supplementary table 4. *CLCC1* rare damaging variants identified in our HC cohort**

| **Mutant Count** | **Chr1** | **Exon** | **cDNA change** | **AA change** | **Mutation type** | **dbSNP** | **GnomAD_exome_ALL** | **GnomAD_exome_EAS** | | **ExAC** | | **1000 Genomes** | | **ESP6500s** | | **Pathoge-nicity^a^** | | |
| --- | --- | --- | --- | --- | --- | --- | --- | --- | --- | --- | --- | --- | --- | --- | --- | --- | --- | --- |
| 1 | 109477297 | 11 | c.G1651A | p.G551S | missense | NA | NA | | NA | | NA | | NA | | NA | | 7 (9) |  |
| 1 | 109479983 | 10 | c. G1099C | p.E367Q | missense | NA | NA | | NA | | NA | | NA | | NA | | 7 (9) |  |
| 2 | 109484081 | 7 | c.G742C | p.E248Q | missense | NA | NA | | NA | | NA | | NA | | NA | | 6 (9) |  |
| 1 | 109486501 | 5 | c. G518T | p.R173L | missense | NA | 4.06E-06 | | 5.80E-05 | | NA | | NA | | NA | | 9 (9) |  |

***Abbreviations:*** AA, amino acids; cDNA, complementary DNA; dbSNP, Database of Single Nucleotide Polymorphism; EAS, East Asian; ESP6500s, NHLBI-ESP project with 6500 exomes; ExAC, Exome Aggregation Consortium; GnomAD, Genome Aggregation Database; HCs: healthy controls; NA, not available; 1000 Genomes, 1000 Genomes Project.

^a^ Genome position was based on GRCh37. RefSeq accession number: NM_ 001048210 for *CLCC1*.
